# Understanding the role of the CB1 toggle switch in interaction networks using molecular dynamics simulation

**DOI:** 10.1038/s41598-021-01767-5

**Published:** 2021-11-16

**Authors:** Sangho Ji, Wonjin Yang, Wookyung Yu

**Affiliations:** 1 Department of Brain and Cognitive Sciences, DGIST, 333 Techno jungang-daero, Daegu, 42988 Republic of Korea; 2 Core Protein Resources Center, DGIST, 333 Techno jungang-daero, 42988 Daegu, Republic of Korea

**Keywords:** G protein-coupled receptors, Computational biophysics, Computational biology and bioinformatics

## Abstract

The cannabinoid receptor 1 (CB1) is a class A G-protein coupled receptor (GPCR) that can exert various effects on the human body through the endocannabinoid system. Understanding CB1 activation has many benefits for the medical use of cannabinoids. A previous study reported that CB1 has two notable residues referred to as the toggle switch, F3.36 and W6.48, which are important for its activation mechanism. We performed a molecular dynamics simulation with a mutation in the toggle switch to examine its role in active and inactive states. We also examined structural changes, the residue–residue interaction network, and the interaction network among helices and loops of wildtype and mutant CB1 for both activation states. As a result, we found that the energetic changes in the hydrogen-bond network of the Na^+^ pocket, extracellular N-terminus–TM2–ECL1–TM3 interface including D2.63–K3.28 salt-bridge, and extracellular ECL2–TM5–ECL3–TM6 interface directly linked to the toggle switch contribute to the stability of CB1 by the broken aromatic interaction of the toggle switch. It makes the conformation of inactive CB1 receptor to be unstable. Our study explained the role of the toggle switch regarding the energetic interactions related to the Na^+^ pocket and extracellular loop interfaces, which could contribute to a better understanding of the activation mechanism of CB1.

## Introduction

According to historical records, people have used cannabis for recreational purposes such as mood enhancement, relaxing muscle, and pain relief from ancient times because of the variety of effects of cannabis^1^. Cannabinoids are also used for medical purposes in many diseases such as dementia, epilepsy, sleep disorders, anxiety disorders, and morning sickness^2^. The problem with using cannabinoids is their various side effects, such as drowsiness, loss of awareness, memory impairment, and hallucination. Therefore, understanding the mechanism of CB1 activation will lead to the safe use of cannabinoids without the side effects. Numerous studies have investigated the molecular interactions of cannabis^3–8^. The cannabinoids, which are the main active components of cannabis, bind to the cannabinoid receptor, which initiate the endocannabinoid signaling pathway. The cannabinoid receptor belongs to the class A G protein-coupled receptor (GPCR) family, which consists of seven transmembrane helices (TMs), three intracellular loops (ICLs), and three extracellular loops (ECLs)^9^. Two subtypes of cannabinoid receptors are currently known: the cannabinoid receptor type 1 and 2 (CB1/CB2). In particular, CB1 is the most abundant GPCR in the brain and central nervous system, which regulates various brain functions and behaviors^10^.

Activation of GPCRs in the G protein coupling pathway is important for the signaling pathway of CB1^11^. A previous study reported the common activation mechanism of the class A GPCR family^12^. When a GPCR is bound to its ligand, it undergoes a conformational change. In this process, some residue interactions such as 3.40–6.48 and 5.51–6.44, as described by Ballesteros and Weinstein numbering^13^, are related to agonist binding, which leads to the rotation of intracellular TM6^11^. During the activation, four residues (2.50, 3.39, 7.45, and 7.49) are closer to the collapsing Na^+^ pocket, which causes TM7 to move toward TM3. The opening of the hydrophobic lock consists of 3.43, 6.40, and 6.41 and the intracellular end of TM6 moves outward and loosens the packing of TM3–TM6^11^. In particular, the outward movement of TM6 is necessary for activating GPCR^14^. Several important molecular interactions in GPCRs change the helical dynamics. Therefore, a study of the interaction network for each residue pair is required to understand GPCR, which is related to its helical dynamics.

The specific motif of GPCRs that modulates the activation of the receptors is called a “molecular switch.” These motifs are affected by GPCR ligands, which include the ionic lock switch, the 3–7 lock switch, the tyrosine toggle switch linked with the NPxxY motif in TM7, and the transmission switch^15^. We focused on the transmission switch in this study, formally called the “rotamer toggle switch”. The rotamer toggle switch motif, referred to as the “toggle switch” is composed of F3.36 and W6.48 in CB1, which links the agonist binding site. A previous study of other GPCR A family receptors reported that the toggle switch drives the movement of neighboring helices. The toggle switches of the β_2_ adrenergic receptor (β_2_AR) and the A_2A_ adenosine receptor (A_2A_AR) mediate the helical movement of TM5 and TM6 through the rearrangement of inter-helical interactions in TM3, 5, and 6^16^. In addition, each loop domain tethered between two neighboring helical domains is affected by helical movements and ligand binding, which is linked to receptor activation^17^. Studying the role of the toggle switch helps to understand the activation of GPCRs. For this reason, it is important to consider the structural changes in the helices and loop regions of CB1.

Before determining the full structure of CB1, a previous study using molecular dynamics (MD) simulation with CB1 provided reliable results for the relationship between the structural motion of helices and the conformational change of the toggle switch using homology modeling for CB1^3,18^. The full structure of human CB1 with the full agonist AM11542 was revealed by X-ray crystallography, which binds to the CB1 structure^4^. Using a similar approach, the inactive state of the CB1 structure was revealed with the inverse agonist taranabant^19^. After defining the full structure of human CB1, several efforts have been made to better understand CB1 dynamics by performing MD simulations, such as capturing the transition moment, ligand binding efficacy, and ligand selectivity^6–8^. However, the role of the CB1 toggle switch from an energetic perspective is not well understood.

According to previous mutagenesis study for the rotamer toggle switch, the mutation of the toggle switch affects the amount of activation of CB1 receptor^3^. Especially, F3.36A mutation showed statistically significant increases in ligand-independent stimulation of GTPγS binding. And W6.48A mutation contributed enhanced agonist activation. Each residue of the toggle switch contributed to the CB1 activation in different ways. However, there is a bottleneck to understand how and why the role of toggle switch residue provides different contribution for the conformational change during CB1 activation. What is certain is that the aromatic contact between F3.36 and W6.48 is an important for the CB1 inactive state^3^. To focus on the role of the toggle switch in the view of structural and energetic level during activation, we performed double mutation for the F3.36 and W6.48 residues.

To determine the role of the toggle switch, MD simulations were performed with wildtype and mutant CB1, in which the toggle switch was converted to alanine. We also compared the conformational and energetic changes between the wildtype and mutant CB1. In addition, the commonly appearing residues were revealed by comparing active and inactive CB1, which show the significant energetic changes after the mutation. They might stabilize the active and inactive conformation of CB1 receptor. As a result, we determined the important interactions among the helices or loops for each activation state. Although there are shared the major component of interaction network, there are some different residue–pairwise interaction of each activation state causes a different conformational change in CB1 against broken and destabilized toggle switch interactions. Our study explained the role of the toggle switch for energetic interactions related to the Na^+^ pocket and extracellular loop interfaces. The discovery of structural details in this study advances our understanding of the role of the CB1 toggle switch. Furthermore, the improved structural knowledge allows us to avoid the side effects of cannabinoids and use cannabinoids wisely and effectively.

## Methods

### Preparation of ligand-bound CB1 complex structures

The active state with THC and the inactive state with taranabant complex structures were used in a previous study^6^, where ICL3 was built using homology modeling with Modeller v9.18^20,21.^ Ligand preparation and docking were performed using the Ligprep^22^, Epik^23–25^, Jaguar^26,27^ and Glide^28,29^ modules in Schrodinger software.

### System building and toggle switch mutation

Membrane Builder in CHARMM-GUI (http://www.charmm-gui.org) was used to build the membrane bilayer^30^. Positioning of proteins in the membrane (PPM) server of the orientations of proteins and membranes (OPM) database was used to determine the spatial positions of CB1 in membranes^31^. Membranes were filled with a 1-palmitoyl-2-oleoyl-sn-glycero-3-phosphocholine (POPC) lipid bilayer. The Tip3p water model^32^ was used to solvate CB1 and the complex. The ion concentration matched 0.15 M NaCl. The system size was 79 Å × 79 Å × 111 Å in the inactive conformation and 88 Å × 88 Å × 116 Å in the active conformation of the CB1 system. The numbers of total atoms for active wildtype, active mutant, inactive wildtype, and inactive mutant system are 94179, 93945, 91139, and 90914, respectively. The numbers of POPC are also 194, 194, 190, and 190, respectively. Furthermore, the active and inactive CB1 mutations were performed using CHARMM-GUI, in which the toggle switch residues F3.36 and W6.48 were changed to alanine.

### Molecular dynamics simulation

All simulations were performed using the AMBER20 MD simulation package^33^ with the CHARMM36m force field^34^. Each system was minimized with the steepest descent of 2500 and a maximum of 2500 conjugate gradient minimization steps. Equilibrations were performed for a total of 375 ps with NVT and NPT ensemble. After equilibration, the simulations were performed for 1 μs under NPT ensemble without any position restraints with a 1.0 nm non-bonded cutoff. The temperature was maintained at 310 K using Langevin dynamics during the simulation, with a collision frequency (γ) of 1.0. The particle-mesh Ewald (PME) method^35^ was applied for long-range electrostatic interactions, and short-range and non-bonded interactions had a 10 Å distance cutoff. The SHAKE algorithm^36^ was used to constrain the bond length of hydrogen atoms, and the time step was 2 fs. For four systems, MD simulations were performed three times so that total 12 μs simulations were done.

### Analysis of the MD simulation trajectory

CPPTRAJ^37^ in the AMBER20 package was used to analyze each trajectory. Root–mean–square deviation (RMSD) and root–mean–square fluctuation (RMSF) calculations were performed for the structure analysis.

#### Separation of helices

Seven TMs of CB1 were manually defined as the regions keeping invariant secondary structures during the simulation for each activation state (Table S1). All trajectories were aligned to their crystal structures as a reference structure for each activation state. Furthermore, TM6 and TM7 were separated into two compartments by proline residues, P358 and P394, respectively, as pivot point.

#### Calculation of the helix angle and RMSD

The angle of each helix was measured between each helix vector and the Z-axis as a membrane normal vector (Fig. S1). The N-terminus and C-terminus endpoints defined each helix vector. The endpoint of each helix was used as the center-of-mass point of 11 backbone atoms of four residues for its terminus, which represented one helical turn of the α-helix. Backbone atoms used only C, Cα, and N atoms, and an inward backbone atom of each terminus in each helix was eliminated. And one gap interval was applied at both termini. All trajectories were pre-aligned to the reference structure. The RMSD for each helix was calculated based on C, Cα, O, and N atoms. The no-fit option was applied for each helix to calculate the amount of shifting and tilting.

#### Constructing an interaction energy network

The MMGBSA^38^ calculation was performed with energy decomposition to determine pairwise residue interactions between every residue. In this analysis, helical interactions with distances of up to four neighboring residues (from residue i to residue i + 4) were eliminated. The interaction energy network was constructed using residue–wise interaction energy and residue–pairwise interaction energy, which were expressed as the depth of color for each residue and the thickness of the interaction bond between two residues. Residue–wise interaction energy was calculated by the summation of residue–pairwise interactions for each residue.

#### Visualization

The interaction energy network was constructed using an in-house Python code with the PYMOL library^39^. To interpret the helical dynamics of CB1, all trajectories were visualized using VMD^40^. For the chord diagram, an additional chord visualization API, Chord Pro^41^, was used. Other visualizations were based on Python 3.6, with the matplotlib 3.3.2 library^42^.

## Results

### Toggle switch-dependent molecular dynamics of the CB1 receptor

**Figure 1:**
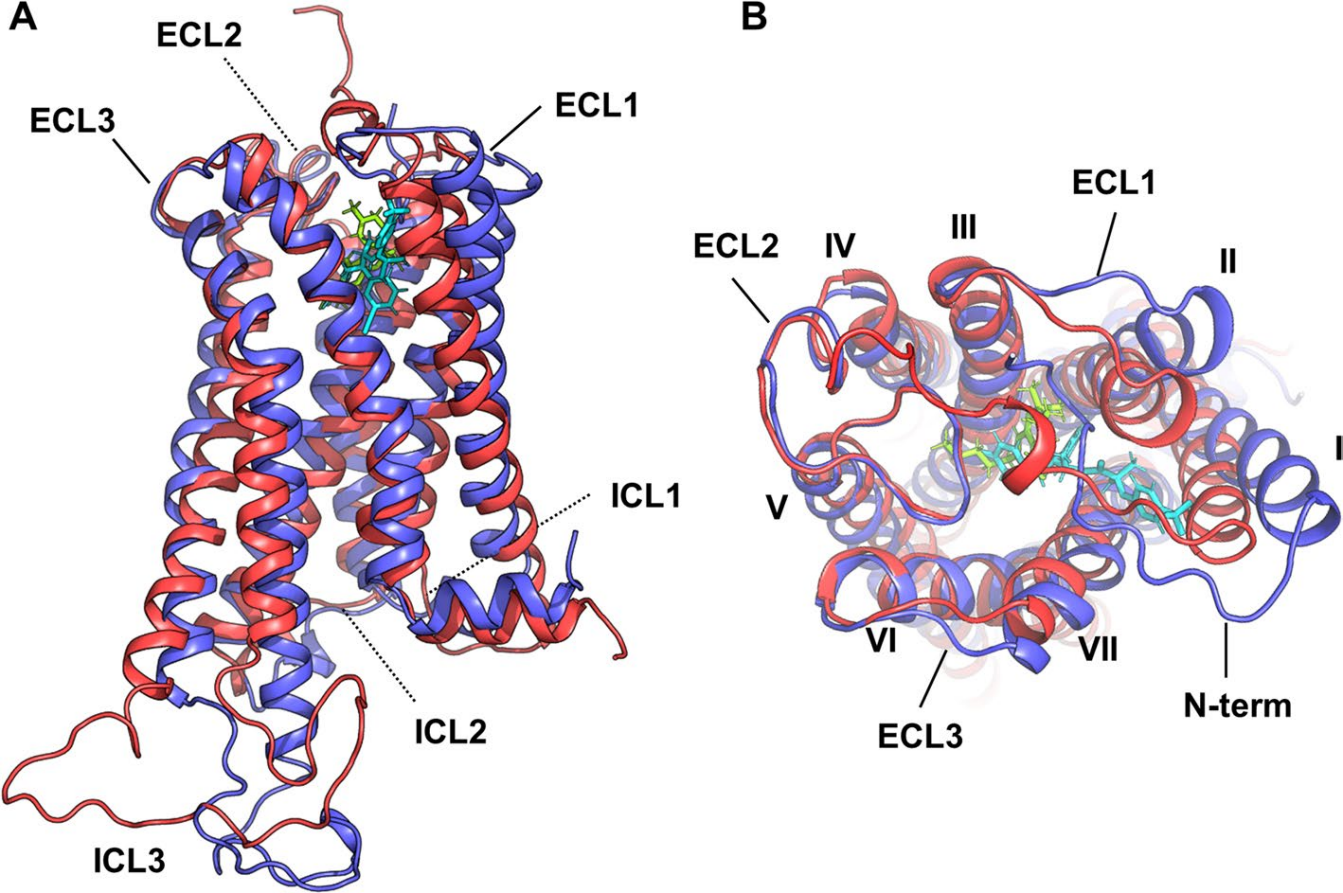
Overall structures of CB1 receptors. Structure of CB1 (**A**) in side view and (**B**) in top view. Colored as: active (red), inactive (blue) of CB1, agonist THC (green) and inverse agonist taranabant (cyan), respectively.

MD simulations were performed to reveal the role of the toggle switch. Both active and inactive structures of the CB1 receptor were prepared from the Protein Data Bank (PDB): 5XRA^4^ (active) and 5TGZ^43^ (inactive). Each prepared CB1 receptor structure was mutated in the toggle switch, identical to F3.36 and W6.48, into alanine to remove π electronic interactions between residues. To maintain the stability of each CB1 receptor, active and inactive structures with appropriate ligands were constructed, such as Δ^9^-tetrahydrocannabinol (THC) and taranabant (Fig. 1). THC is the natural agonist of CB1 which is well kwon and the most frequently used agonist for CB1 effects on our brain and taranabant is also well-known inverse agonist of CB1. All wildtype and mutant structures with membranes were constructed using the CHARMM-GUI web-server^30^. After preparing the CB1-ligand complex structures, MD simulations were performed using AMBER^33^. The calculated trajectories were aligned to each X-ray crystal structure as the reference in accordance with each activation state.

**Figure 2:**
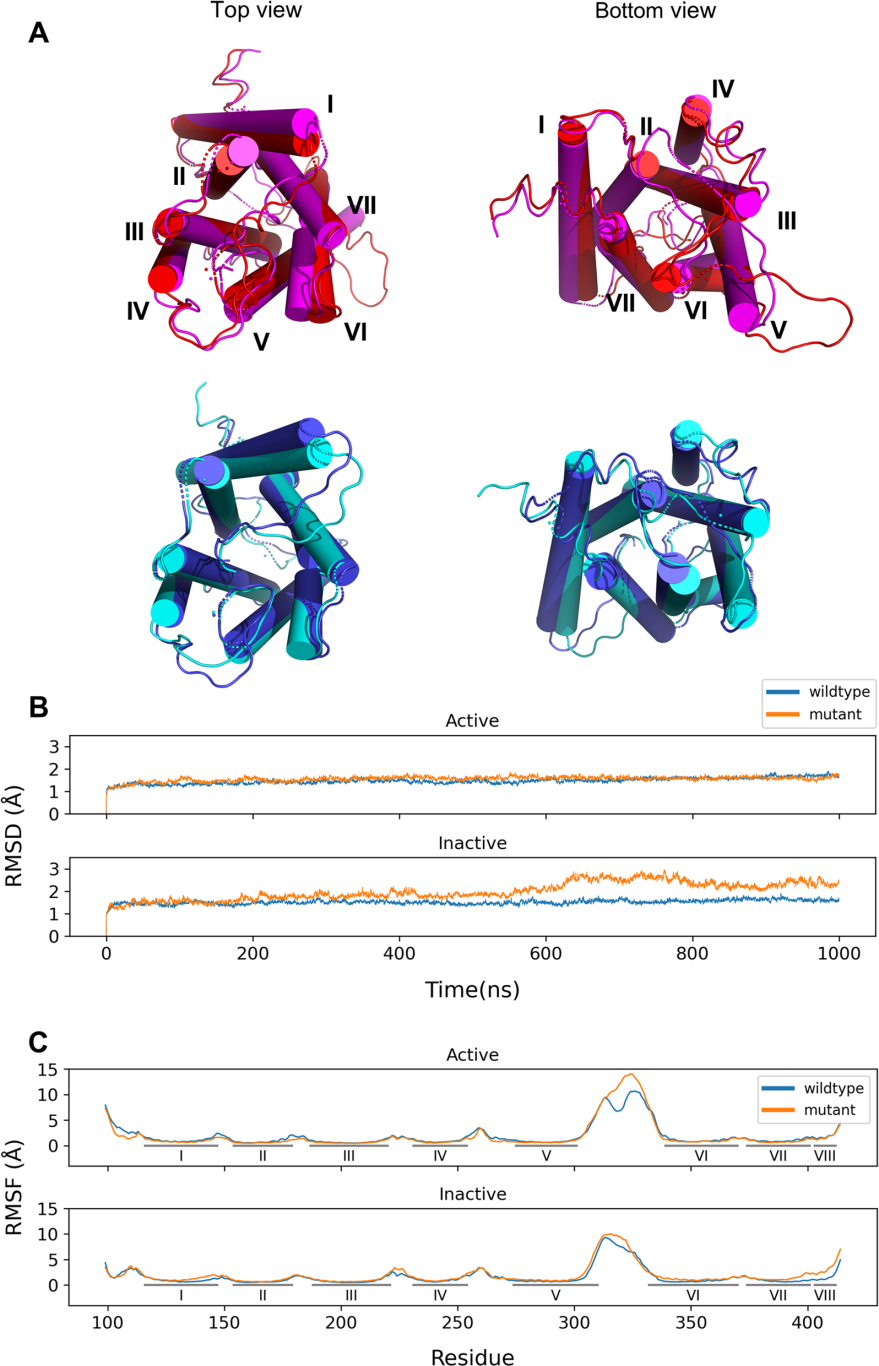
Structural and dynamical differences between wildtype and mutant CB1 for each activation state. (**A**) The comparisons of averaged structure between wildtype and mutation for each activation state in CB1. Colored as active wildtype (red)/mutant (magenta) and inactive wildtype (blue)/mutant (cyan). (**B**) The average of RMSD and (**C**) RMSF for three MD trajectories between wildtype (blue) and mutant (orange) CB1 for each activation state are presented. The RMSD is calculated without loop regions.

First, we examined the structural differences between the wildtype and mutant for the active and inactive state of the CB1 receptor (Fig. 2). The averaged structure was calculated for each trajectory. The averaged cylindrical helices showed structural differences. In active and inactive states, TM1, 6, and 7 were slightly changed depending on the toggle switch mutation (Fig. 2A). The active state showed the inward position of TM1 and a small change in the other helices compared with the inactive state, a tendency that has already been reported in a previous study^4^ (Fig. S2). We also calculated the RMSD for the CB1 receptor without the loop regions (Fig. 2B). During the simulation, the RMSD showed that the CB1 structure remained stable until the end of the simulation, which guarantees further reliable analysis. Further RMSF analysis showed an increased fluctuation of TM7 and TM8 only in the mutant inactive state (Fig. 2C). This different fluctuation pattern might have occurred because of the mutation of the toggle switch.

### The change of helical orientations by toggle switch mutation

**Figure 3:**
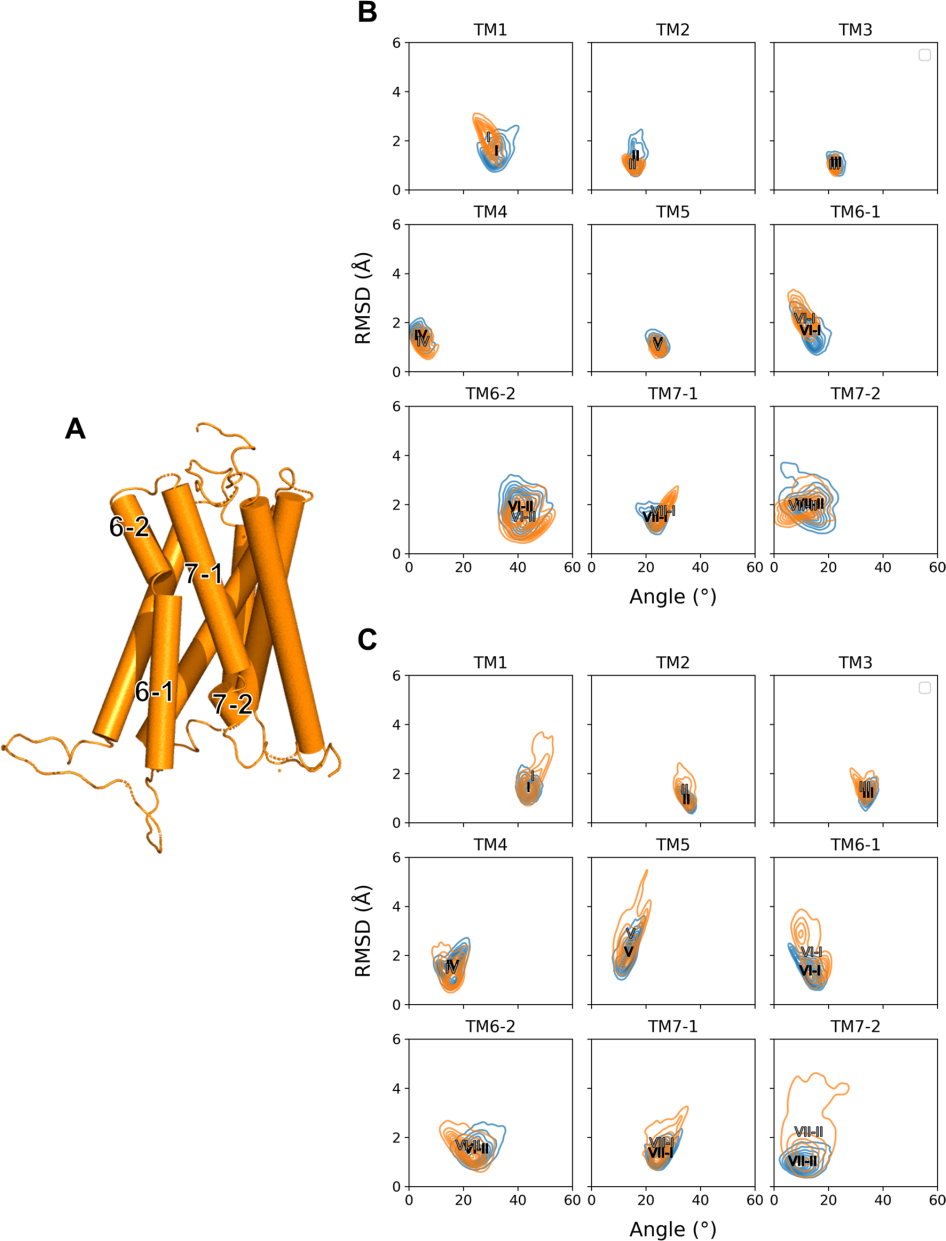
The angle-RMSD distribution for each helix. (**A**) Schematic helical compartments of CB1. Especially, TM6 and TM7 are separated into two compartments by its proline residue as pivot point. (**B**, **C**) The angle orientation and RMSD of each TM on both wildtype (blue) and mutant (orange) CB1 are plotted for (**B**) active and (**C**) inactive states. The two-dimensional density plot of angle and RMSD are shown over three trajectories for each state. The average point for each TM is marked as its TM index in wildtype (black) and mutant (gray).

To elucidate the effects of toggle switch mutation on the CB1, we measured the angle distributions and RMSD for each helix (Fig. 3). The mutation of the toggle switch causes macromolecular changes in both activation states, such as the shift or rotation of helices. In particular, the intracellular topology of the CB1 helices, might be important for its function such as G protein activation. We calculated the helical angle from the helical vector and the Z-axis as the membrane normal vector (Fig. S1). TM6 and TM7 of the CB1 receptor are normally shown as bent shapes. Thus, we separated TM6 and TM7 into two compartments with the proline residue as a bent point for a detailed understanding. This separation is illustrated in Fig. 3A.

All angle and RMSD data for wildtype and mutant active and inactive CB1 are presented (Fig. 3B,C). Some helices showed several interesting features between the wild type and the mutant. The RMSDs of TM1, 5, 6, and 7 in the inactive state increased after mutation, suggesting tilting, or shifting. The RMSD of TM1 and TM6-1 in the active state was also slightly increased after mutation. However, the difference is not significant compared with the inactive CB1 receptor. The distribution of RMSD of TM7 region were not different. Almost all helix angle distributions in the CB1 structure were unchanged. And the helical angles for TM1, 6–1, and 7–1 in the active state and TM1 in the inactive state were a little changed, suggesting small reorientation of the TMs. As a result, the dynamic changes in TM1, 6 and TM7 were noticeable. All the time-evolution data of the activation-dependent helical dynamics are presented in Figs. S3 and S4. The results implied that TM6-1 might be related to TM6-2, TM5, and TM7-2. Sequentially, TM1 also associated the perturbation of TM5, 6 and 7 because the RMSDs for TM1, 6, and 7 in the inactive state simultaneously fluctuated at the same time, after 500 ns in the simulation.

### Analysis of interactions among CB1 helices and loops

The measurement of helical orientations showed several significant features for interpreting the mutation effects of the toggle switch. The interaction energy between each helix of CB1 was calculated by MMGBSA analysis^38^ to understand the mechanism of helical interactions. In the MMGBSA calculation, energy decomposition was applied, which provided interactions for residue–residue pairs. Because the main interaction was a hydrogen bond interaction in the α-helix and an adjacent interaction between two neighboring residues, we ignored all helical interactions and adjacent interactions within the four residues.

**Figure 4:**
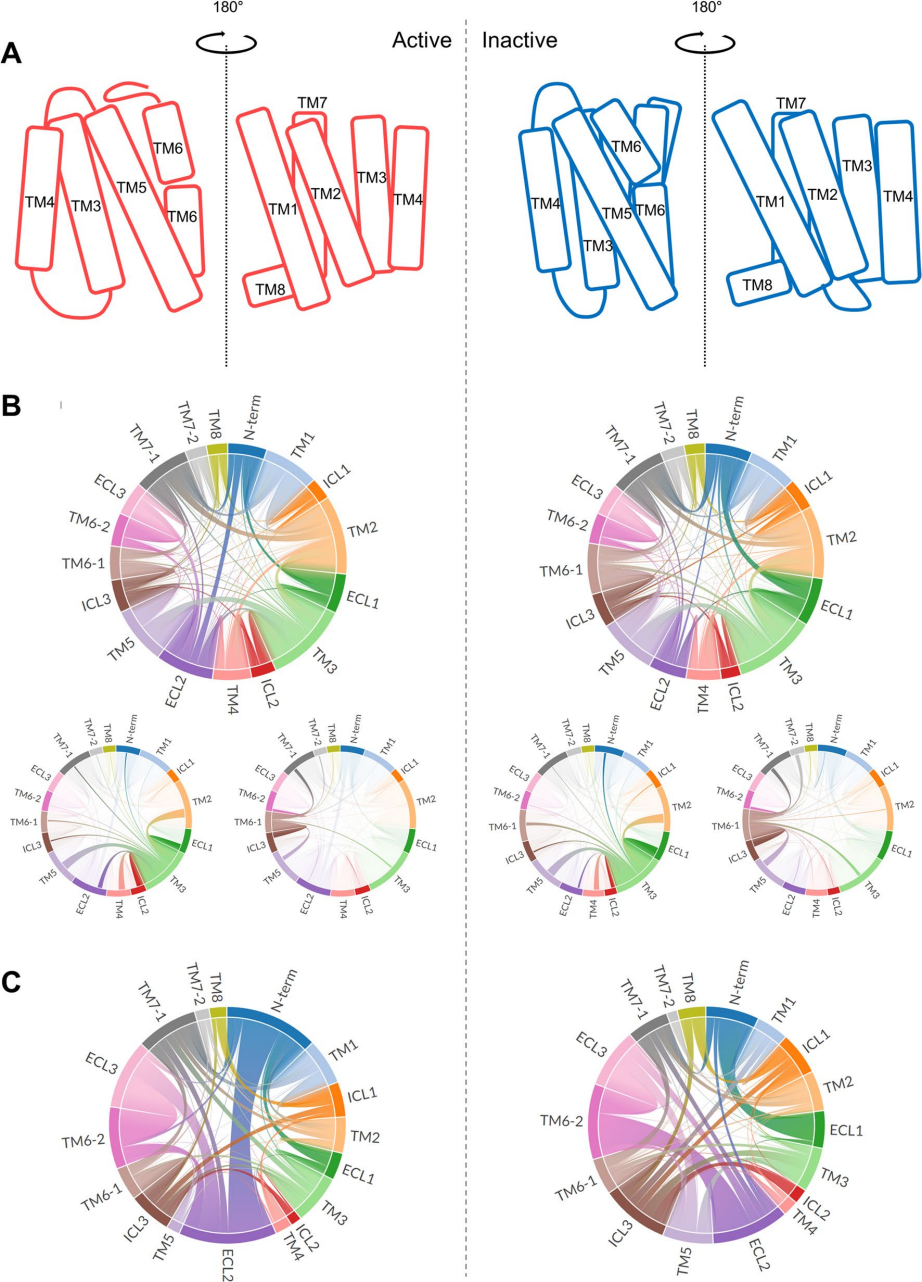
The interaction energies among helices and loops of CB1. (**A**) Simplified relative position of helices for active and inactive CB1. (**B**) The pairwise interaction among helices and loops of wildtype CB1. Especially, the pairwise interactions for TM3 and TM6 in which the toggle switch is located are separately presented. (**C**) The energetic changes for pairwise interaction after the mutation.

To better understand the helical interactions, the structural information of active and inactive CB1 is presented (Fig. 4A). To interpret the interaction between each helix and loop, we calculated the by-group contribution of the interaction energy for each helix and loop in both activation states (Fig. 4B). In particular, the interactions for TM3 and TM6, which include toggle switch residues, were visualized. The results showed that TM2–TM3, TM2–TM7, and TM3–TM5 interactions were stronger than other inter-helical interactions. This finding shows that TM3 was already sufficiently stabilized in the interaction network of wildtype CB1. In contrast, TM6 showed relatively weak interactions with the other regions. However, the interaction energy difference between the wild-type and mutant showed that TM6, especially TM6-2, and the neighboring ECL regions changed more in both activation states (Fig. 4C). TM3 appears to be more effectively stabilized by its neighbor interactions than TM6 when the toggle switch is mutated.

### The residual interaction network around the Na^+^ pocket and extracellular loop regions

**Figure 5:**
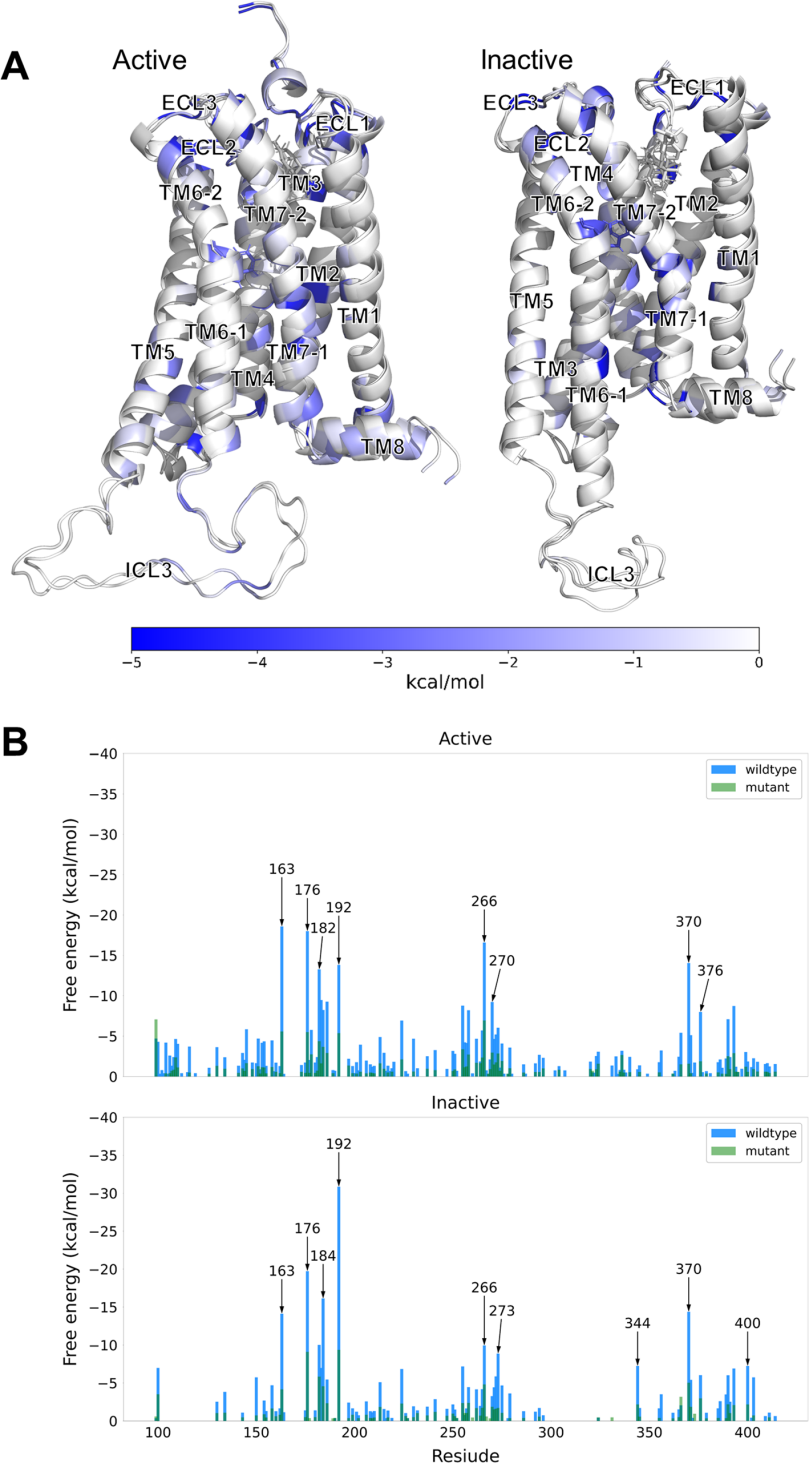
The comparison of residual interaction energies between wildtype and mutant CB1. (**A**) For each activation state, all residues are colored as blue according to their residual interaction energies. The CB1 structures are used the structures after energy minimization. (**B**) The significant residues between wildtype (blue) and mutant (green) for each activation state.

For a more detailed understanding of CB1 interactions, the residual interaction energy was visualized as a network of CB1 (Fig. 5A). The residual interaction energy was calculated from the sum of pairwise interactions for each residue. The specific residues that have strong interaction energy with other residues are marked with deeper blue colors. We determined the specific residues strongly affected by the toggle switch mutation from an energetic perspective (Fig. 5B). Among them, D163, D176, R182, D184, K192, and D266 were commonly appeared in both activation states. These residues showed strong interactions with both the wildtype and mutant.

Furthermore, we analyzed the by-residue energetic contributions for residues D163 (D2.50), D176 (D2.63), R182, D184, K192 (K3.28), and D266 located in TM2, ECL1, TM3, and ECL2, respectively. D2.50 is a well-known member of the Na^+^ pocket and is critical for sodium-dependent effects^12^. D2.63 and K3.28 are also crucial key composition of CB1 receptor activation by forming salt bridge^44^. We tried to check their interaction networks, including their neighboring residues. We expressed the pairwise interaction as the thickness of the bond in the interaction network (Figs. 8, 9).

**Figure 6:**
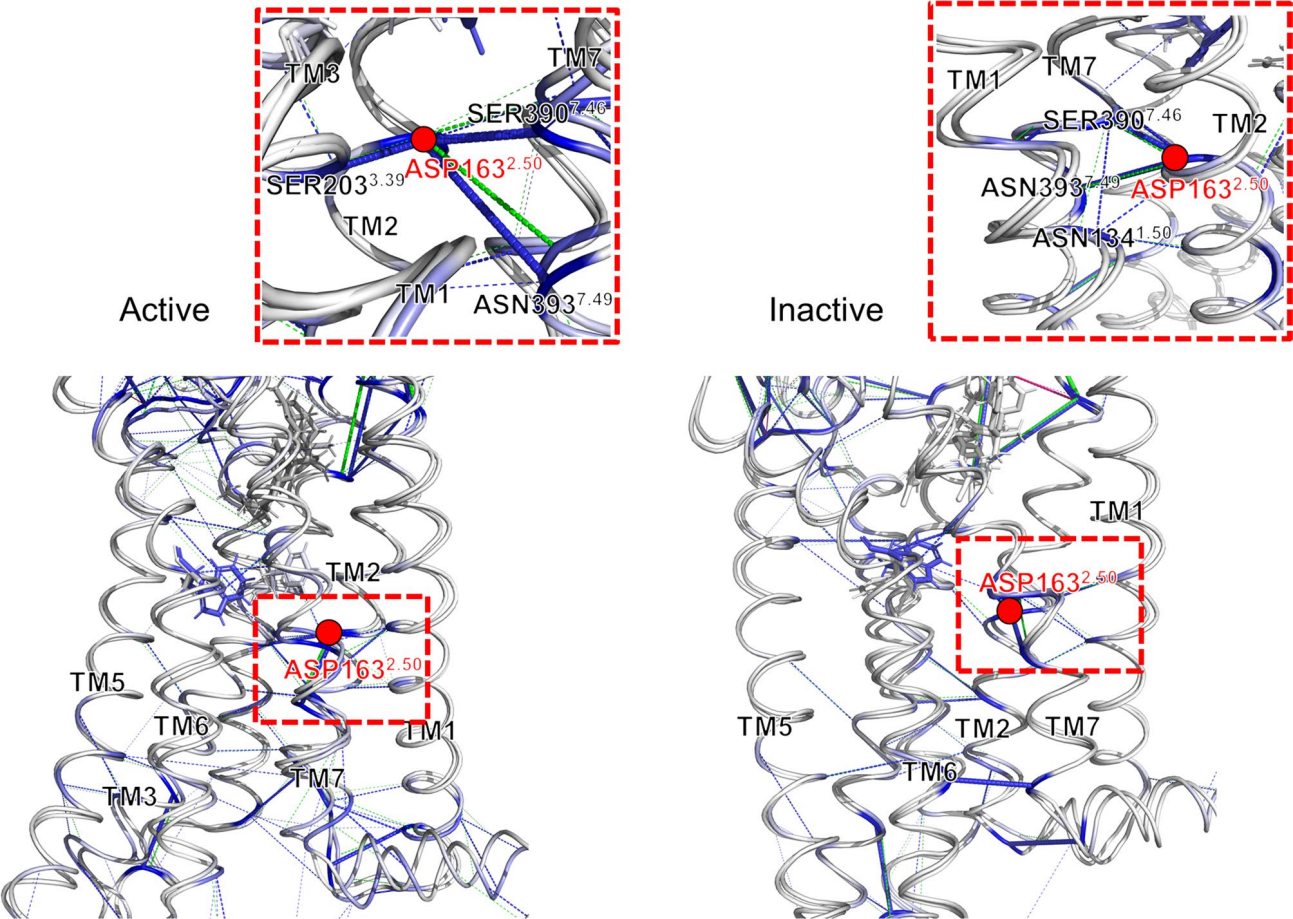
The hydrogen bond network of Na^+^ pocket around D163 (D2.50). The residue pairwise interactions are visualized as bonds for wildtype (blue) and mutant (green) CB1. And each thickness of bond is the strength of pairwise interaction energy. The locations of D163 (D2.50) are marked as red circles. Especially, the hydrogen bond networks of Na^+^ pocket in active and inactive CB1 are zoomed in.

First, we checked the neighbor interaction network around the D163 residue in the Na^+^ pocket (Fig. 6). In both CB1 receptor activation states, the D163 residue interacts with two neighboring residues in TM7, D390 (D7.46), and N393 (N7.49). In the active state, D163 has an additional interaction with S203 (S3.39). In the inactive state, D163 weakly interacts with N134 (N1.50) instead of S203. Interestingly, the compositions of the interaction network around D163 differed according to the activation states of CB1. This difference causes a change in the geometrical location of the interaction network for D163, which is more proximal to the toggle switch and has more additional interactions with TM3 and TM6 in the active state (Fig. 6). In the active state, TM3 is more likely to be stabilized by the proximal Na^+^ pocket. We checked whether the interaction network formed hydrogen bonds. Some hydrogen bonds in the Na^+^ pocket are weak or broken after the mutation (Fig. S5). In particular, this tendency is more apparent in the inactive state than in the active state. The interaction energy among these hydrogen bond clusters on both activation states was smaller in the mutant than in the wildtype. It seems that the hydrogen bond cluster might play a key role in the energetic stabilization of CB1 related to toggle switch interactions. Some energetic compositions should compensate for the fluctuation of TM3/6 to maintain the stability of CB1.

**Figure 7:**
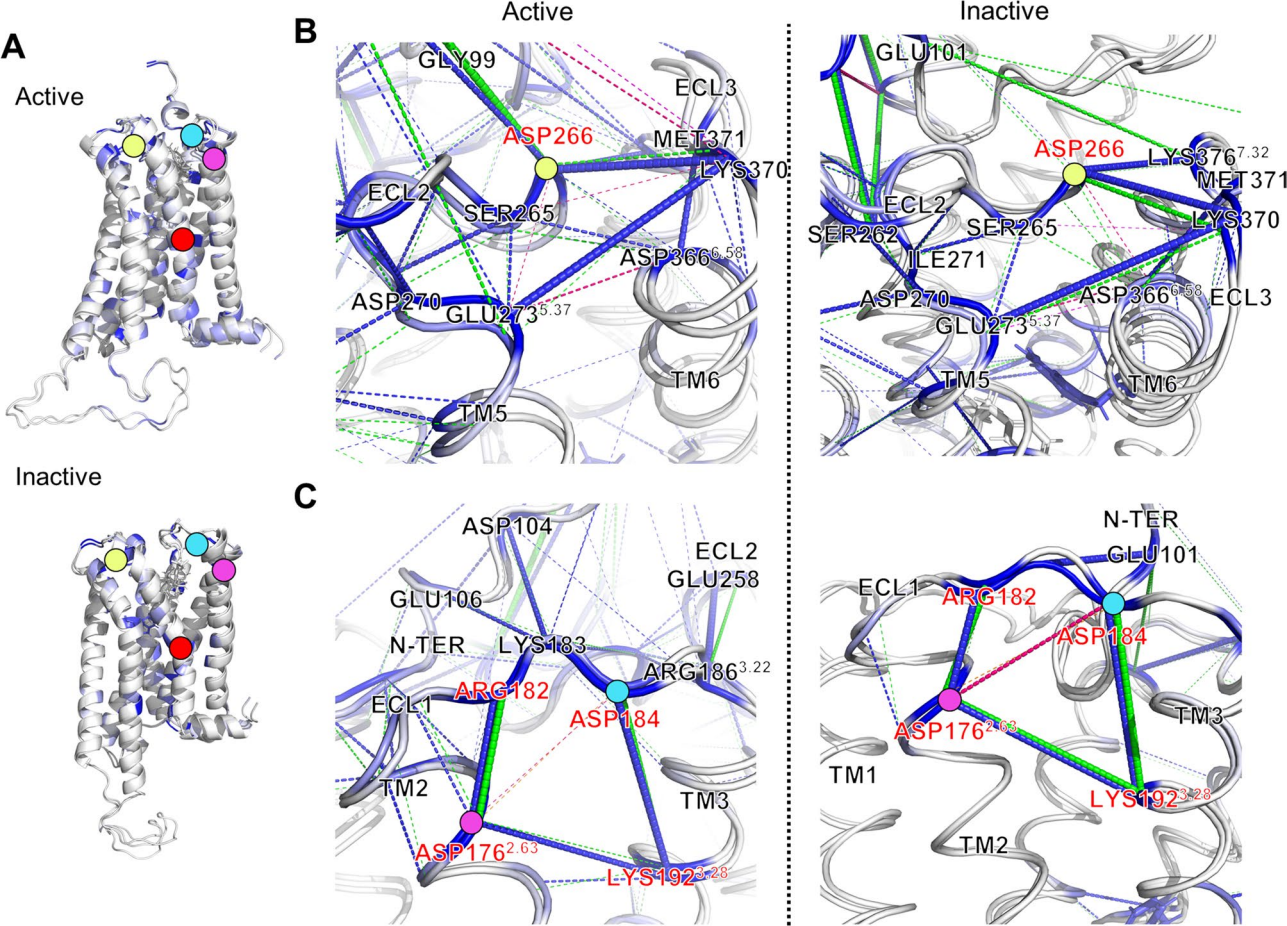
The interaction network of extracellular loop interface of CB1. (**A**) The location of D176 (D2.63) (pink), D184 (cyan), D266 (lemon), and D163 (D2.50) (red) in active and inactive CB1 structures. (**B**) The interaction network among ECL2, TM5, TM6, and ECL3 of active and inactive CB1. (**C**) The interaction network among N-terminus, TM2, ECL1, TM3, and ECL2 of both activation states. Attractive interactions are colored according to wildtype (blue) and mutant (green) CB1. Repulsive interactions (only mutant CB1 appeared) are also colored wildtype (magenta) and mutant (orange). Each thickness of bond is the amount of pairwise interaction energy.

Next, the interaction network of extracellular loops around D366 and D266 was examined (Fig. 7). The position of each residue was marked with a different color (Fig. 7A). In both activation states, some residues such as S265, D266, E273 (E5.37), D366 (D6.58), and K370 have strong interactive connections (Fig. 7B). D266–K370, D366–M371, and E273–K370 strong interaction pairs appeared in the interaction network of the CB1 mutant. These strong interactions disappeared after the mutation. From previous helical dynamics, TM5, in which E273 is located, TM6-2, in which D366 is located, are not significantly changed in RMSD in inactive state (Fig. 3B,C). This finding implies that the long-range charge–charge interaction between TM5 and ECL3 vanishes after the mutation without TM5/6 movement, and only the ECL2–ECL3 interaction remains in active CB1 receptor. In opposite, the movement of TM5, 6 and 7 in inactive CB1 receptor is observed in RMSD (Fig. S4). It might be influenced in the N-terminus region. The interaction network among ECL2, ECL3, TM6-2, and N-terminus are significantly changed in energetic level after the mutation (Fig. 4C). Especially, N-terminus region is strongly connected with ECL2–ECL3 in active state, in opposite, it is connected with ECL1 in inactive state (Fig. 4B). Also, N-terminus is connected with ECL2–ECL3 in active and ECL1 in energetic change after the mutation (Fig. 4C). The residual interaction energy analysis shows that there are more strong interactions in the N-terminus region of inactive CB1 receptor (Fig. 5B). The N-terminus region of inactive CB1 receptor might not efficiently stabilize its ECL2–TM5–ECL3–TM6 interaction network.

We examined another interaction network including the salt-bridge compositions, D176 (D2.63) and K192 (K3.28) (Fig. 7C). D176 residue composes a strong interaction as same as the D163 residue in the Na^+^ pocket (Fig. 5B). As mentioned above, N-terminus residues interacts with ECL1 region and the ECL1 is strongly connected with D176 and K192. Interestingly, the neighboring residues D176, R182, D184, and K192 residues are significantly reduced in interaction energy level by the toggle switch mutation. It might be caused by breaking the hydrogen bonding of Na^+^ pocket in the same TM region or the direct effect of the toggle switch mutation. Thus, the toggle switch mutation directly or indirectly makes the conformation of inactive CB1 receptor to be unstable though Na^+^ pocket, extracellular N-terminus–TM2–ECL1–TM3, and ECL2–TM5–ECL3–TM6 interaction network.

## Discussion

**Figure 8:**
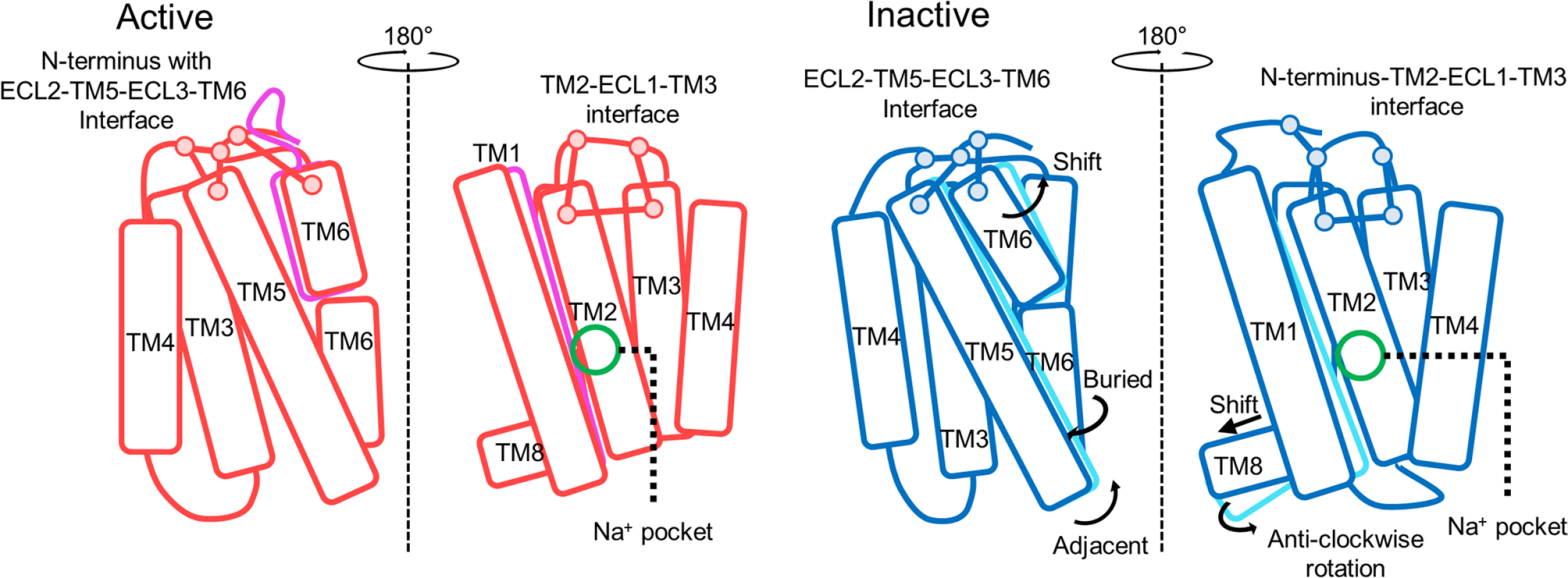
Schematic figure of dynamical changes and inter-helical interaction changes after the toggle switch mutation in CB1. The active and inactive of wildtype CB1 structures are colored as red and blue and of mutant CB1 are magenta and cyan, respectively. Pairwise interactions are presented as solid lines, which are decreased after the mutation. Especially, the position of Na^+^ pocket is marked as green circle.

Many internal interactions and molecular switches influence the dynamic changes in CB1. Toggle switch mutations led to small changes in TM3 and TM6 so that neighboring helical domains and loop domains were influenced, leading to further changes. Significant interactions between each helix in the interaction network were observed in both activation states (Fig. S6). The inter-helical interactions of the active and inactive states were different, as were the structural details of active and inactive CB1. These differences account for the energetic difference of CB1 during each activation state, which seems to lead to different changes in the angle and RMSD of helices (Fig. 3B,C). Based on these conformational changes and the energetic analysis of the interaction network, we determined the role of three interaction clusters: the Na^+^ pocket, extracellular N-terminus–TM2–ECL1–TM3 interface and extracellular ECL2–TM5–ECL3–TM6 interface, as shown in Fig. 8. Our results showed that the significant movements for TM1, 5, 6, and 7 appeared in inactive state of CB1 after the toggle switch mutation. Other TM regions were only slightly changed compared to the above three regions (Figs. 3 and S4). Simultaneously, the slight changes in TM3 and TM6 affected the neighboring TM regions and extracellular loops in the interaction networks (Fig. 4). TM3 loosened the hydrogen bonds in the Na^+^ pocket and changes in N-terminus–TM2–ECL1–TM3 interface in network analysis and TM6 drove the dynamic changes in extracellular ECL2–TM5–ECL3–TM6 interface (Figs. 5, 6, 7).

**Figure 9:**
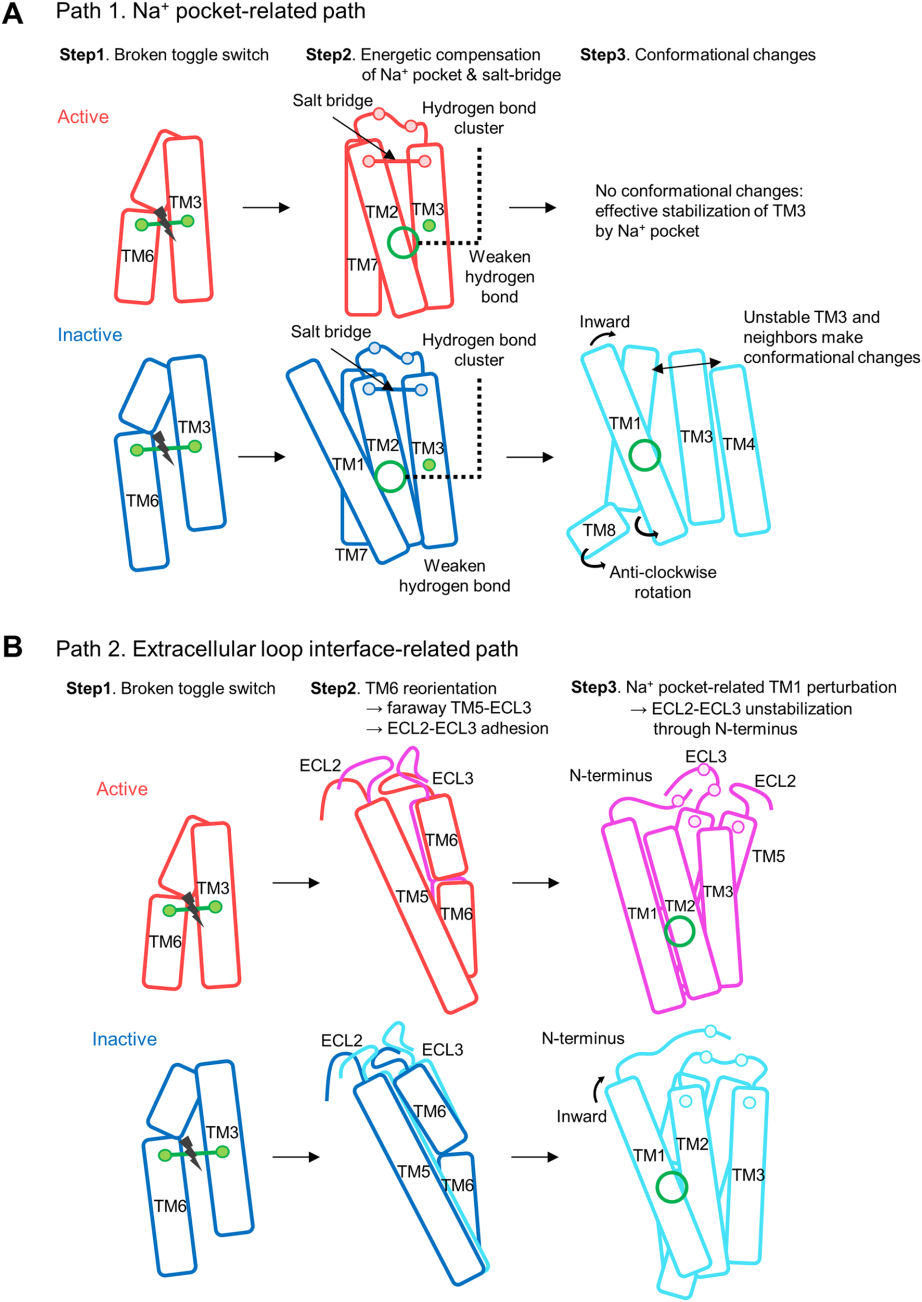
The effects of toggle switch mutation of CB1 related with Na^+^ pocket and extracellular loop interfaces. (**A**) The role of Na^+^ pocket and D2.63–K3.28 salt bridge against the destabilization of TM3–TM6 interaction by the mutation of toggle switch. (**B**) The structural changes by anti-clockwise rotation and outward shifting of extracellular TM6. Consequently, the conformational change of interaction network among ECL2, TM5, TM6, ECL3, and neighbor N-terminus of CB1 receptor is induced.

Several TM regions could form a hydrogen-bond network that is conceivably involved in the activation mechanism of the GPCR A family. This hydrogen bond network consists of TM 1, 2, 3, 6, and 7 obtained from the rhodopsin and β_2_-adrenoceptor^45^. In the A_2A_AR of the GPCR A family, a sodium ion and water molecules are bound to the hydrogen bond network, of which the conserved allosteric binding site, called the Na^+^ pocket, includes several residues such as N1.50, D2.50, S3.39, W6.48, N7.45, N7.49, and Y7.53^46^. N1.50 of TM1 is a conserved residue, which is involved in a structural water-mediated hydrogen-bonding network. The N1.50 residue might contribute to the receptor activation process by driving the movements of TM3, 5, and 7^45^. Also, previous mutagenesis studies have implicated a conserved D2.50 residue in TM2, which is critical for sodium-dependent effects. The mutation of D2.50 into uncharged residues inhibits agonist-dependent signaling in some GPCRs^47,48^. Similarly, CB1 has a similar hydrogen bond network around D2.50. However, the composition of these hydrogen bond networks during each activation state was slightly different. Depending on the composition of the hydrogen bond cluster, the angular and structural changes of the helices are significantly different for each activation state (Fig. 9A). Our results showed that the strength of the hydrogen bonds in the cluster were weaker after the mutation. It seems that the hydrogen bond cluster helps maintain the stability of CB1 together with the toggle switch. In detail, two commonly appearing hydrogen bond linkages, D2.50–S7.46 and D2.50–N7.49, were conserved, but D2.50–S3.39 appeared only in the active state. D2.50–N1.50 appeared only in the inactive state (Fig. S5). This implies that the hydrogen bond cluster in the active state strongly seizes TM3 through S3.39, but the inactive state does not. This difference causes a different mutation effect, such as the fluctuation of neighboring helices around TM3 (Fig. 9A). In addition, the extracellular inter-connection between TM2 and TM3 is influenced by the toggle switch mutation through another way. Especially, the network composed with D2.63, D184 in ECL1, and E101 in N-terminus had a crucial role to stabilize the dynamics of TM2 and TM3 in inactive CB1 receptor (Fig. 7C). According to the previous study, forming the D2.63–K3.28 salt-bridge has a crucial role in constitutive activity of CB1 receptor^44^. Our results supported that this D2.63–K3.28 salt-bridge can be broken by the toggle switch mutation. This situation interrupts stabilizing the inactive conformation of CB1 receptor.

The interaction networks of the extracellular loops and helix regions are complicated. The long-range charge-charge interaction between E273 in TM5 and K370 in ECL3 appeared in wildtype CB1. When the toggle switch is mutated, the ECL2–ECL3 interaction remains, but TM5–ECL3 does not (Fig. 7B). A previous study of rhodopsin receptors reported sequential conformational changes from the outward movement of ECL2 to the changes of TM5 and the inward movement of the TM6–ECL3–TM7 interface during activation^49^. The TM4–ECL2–TM5 unit is related to TM5 and the extracellular ends of TM6 and TM7 in the inactive conformations of rhodopsin^49^. The ECL2 loop can also be affected by TM3 through disulfide bonds linking with conserved C3.25 in the GPCR A family^17^. Based on the above relationship, the rearrangement in the hydrogen-bonding networks, consisting of ECL2 with the extracellular ends of TM4, 5, and 6, plays a crucial role in activation^49^. Thus, the toggle switch mutation can influence ECL2 and ECL3 with TM5 and TM6 during CB1 activation. These effects were observed in our CB1 mutation simulation (Figs. 4C and 9B). In addition, the attractive long-range charge-charge interaction E273–K370 helps to stabilize the hydrogen-bond interactions in the ECL2–TM5–ECL3–TM6 interface. The conformational changes of TMs of CB1 is not equal between the active and inactive states. In the inactive state, the RMSD of TMs was significantly changed after the mutation (Fig. 3C). It is consistent with the well-known facts that the F3.36/W6.48 interaction is a key for the maintenance of the CB1 inactive state. The CB1 toggle switch limits the relative mobility of the cytoplasmic ends of TM3 and TM6, especially in the inactive state, and acts as an “ionic lock”^3^. The mutation of F3.36 to a smaller residue, which does not preserve a steric block to a conformational change in W6.48, leads to increased ligand-independent activation of CB1. It can cause anti-clockwise rotations of extracellular TM3 and TM6^3^. In our simulation, CB1 showed a similar significant change in inactive CB1 receptor with TM6-1 outward movement (Fig. 3C). In summary, the TM5–ECL3 interaction disappeared, and the TM4–TM5 interaction spontaneously reconfigured. This causes an alteration of the TM5–ECL3 interaction into the ECL2–ECL3 interaction and significant change of inactive CB1 receptor with the outward movement of intracellular TM6 (Fig. 9B).

## Conclusions

Understanding the activation mechanisms of GPCRs, including the GPCR A family, is very important in biological and pharmacological fields. Many previous studies have reported that molecular switches, including the rotamer toggle switch, contribute to the activation mechanism. The aromatic interaction in the toggle switch modulates the behavior of neighboring TMs and loops, similar to an ionic lock in the inactive state of class A GPCRs. In our study, we revealed that the hydrogen-bond network of the Na^+^ pocket, forming D2.63–K3.28 salt-bridge of N-terminus–TM2–ECL1–TM3 interface, and extracellular ECL2–TM5–ECL3–TM6 interface contribute to the stability of CB1. There clusters are directly linked to the toggle switch. Our interaction network analysis revealed interesting features in CB1. For example, the hydrogen-bond cluster in the Na^+^ pocket differs between the active and inactive states of CB1. It causes a different efficiency of energetic compensation for the extinct toggle switch interaction. Previous studies suggested many other specific interactions appear only in the inactive state, such as the ionic lock-like toggle switch interaction and a 3–7 salt bridge lock, among other interactions. These interactions cause the specific behavior of CB1, such as the anti-clockwise rotation of extracellular TM5–ECL3–TM6, TM1, and TM7, which strongly appeared in the inactive state. Together with these interactions, the rotamer toggle switch control the stability of CB1 receptor during the activation. According to our finding,  Na^+^ pocket, N-terminus–TM2–ECL1–TM3 interface, and ECL2–TM5–ECL3–TM6 interface help to the conformational stabilization of CB1 with the toggle switch.

## Supplementary Information

Supplementary Information.
