# Supplementary material for: Understanding the role of the CB1 toggle switch in interaction networks using molecular dynamics simulation
Source: Sci Rep. 2021 Nov 16;11:22369. doi: 10.1038/s41598-021-01767-5 (PMC8595625; doi:10.1038/s41598-021-01767-5)
Supplement: Supplementary file 1 — Supplementary Information. [file 41598_2021_1767_MOESM1_ESM.pdf]

*Supplementary Information for*

# Understanding the role of the CB1 toggle switch in interaction networks using molecular dynamics simulation

Sangho Ji[1, †] , Wonjin Yang[1, †], Wookyung Yu[1, 2, *]

1 Department of Brain and Cognitive Sciences, DGIST, 333 Techno jungang-daero, Daegu, 42988, Republic of Korea

2 Core Protein Resources Center, DGIST, 42988, 333 Techno jungang-daero, Daegu, Republic of Korea

† These authors contributed equally to this work.

* Author to whom correspondence should be addressed.

Wookyung Yu : wkyu@dgist.ac.kr

**This PDF file includes:**

Tables S1
Figures S1 to S6

**Table S1. Residues of secondary structure in CB1**

| Secondary Structure | Active state | Inactive state |
|---|---|---|
| N-terminal | 1-16 | 1-16 |
| Transmembrane helix 1 | 17-48 | 17-48 |
| Intracellular loop 1 | 49-54 | 49-54 |
| Transmembrane helix 2 | 55-80 | 55-80 |
| Extracellular loop 1 | 81-87 | 81-88 |
| Transmembrane helix 3 | 88-121 | 89-122 |
| Intracellular loop 2 | 122-131 | 123-131 |
| Transmembrane helix 4 | 132-155 | 132-155 |
| Extracellular loop 2 | 156-175 | 156-174 |
| Transmembrane helix 5 | 176-202 | 175-211 |
| Intracellular loop 3 | 203-239 | 212-232 |
| Transmembrane helix 6 | 240-271 | 233-271 |
| Extracellular loop 3 | 272-274 | 272-303 |
| Transmembrane helix 7 | 275-313 | 304-313 |
| C-term | 314-316 | 314-316 |

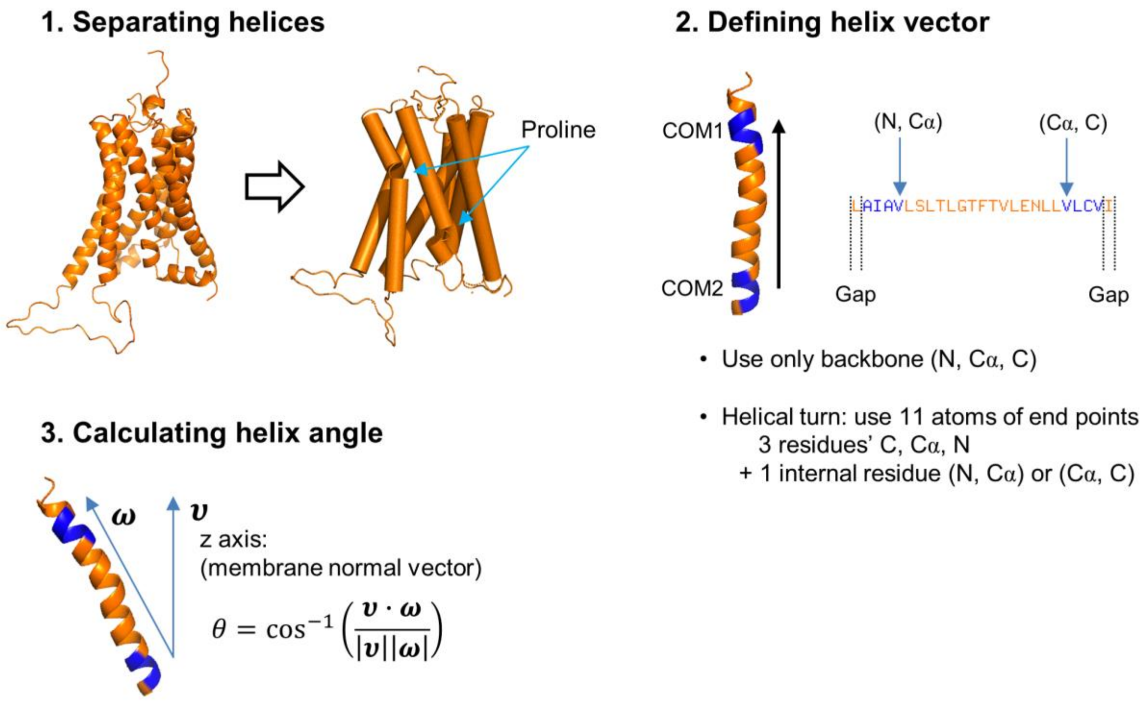


**Fig. S1. The process of calculating helix angle.** All trajectories are aligned to its X-ray crystal structure.

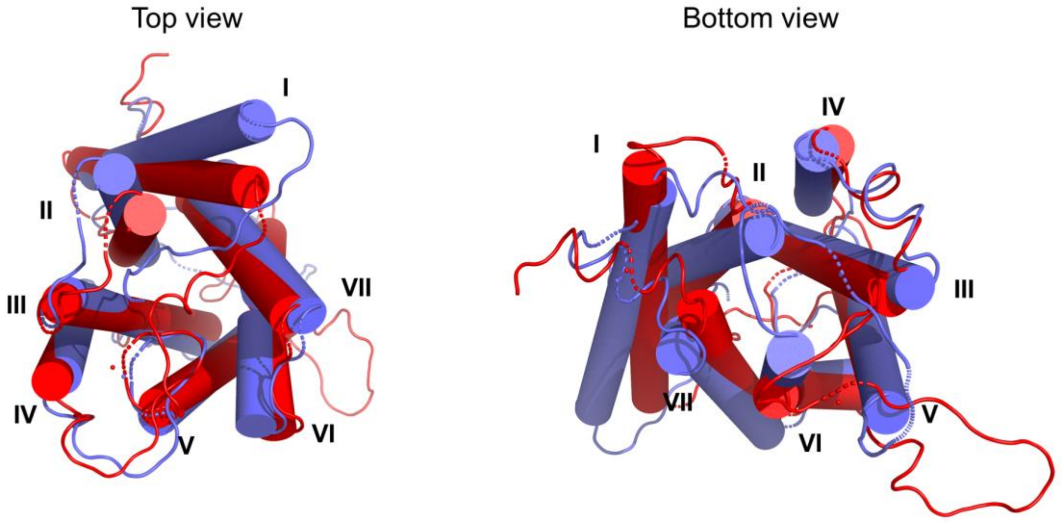


**Fig. S2. The comparison of averaged structure between active and inactive state of CB1.** The active and inactive states of CB1 are shown in red and blue color, respectively.

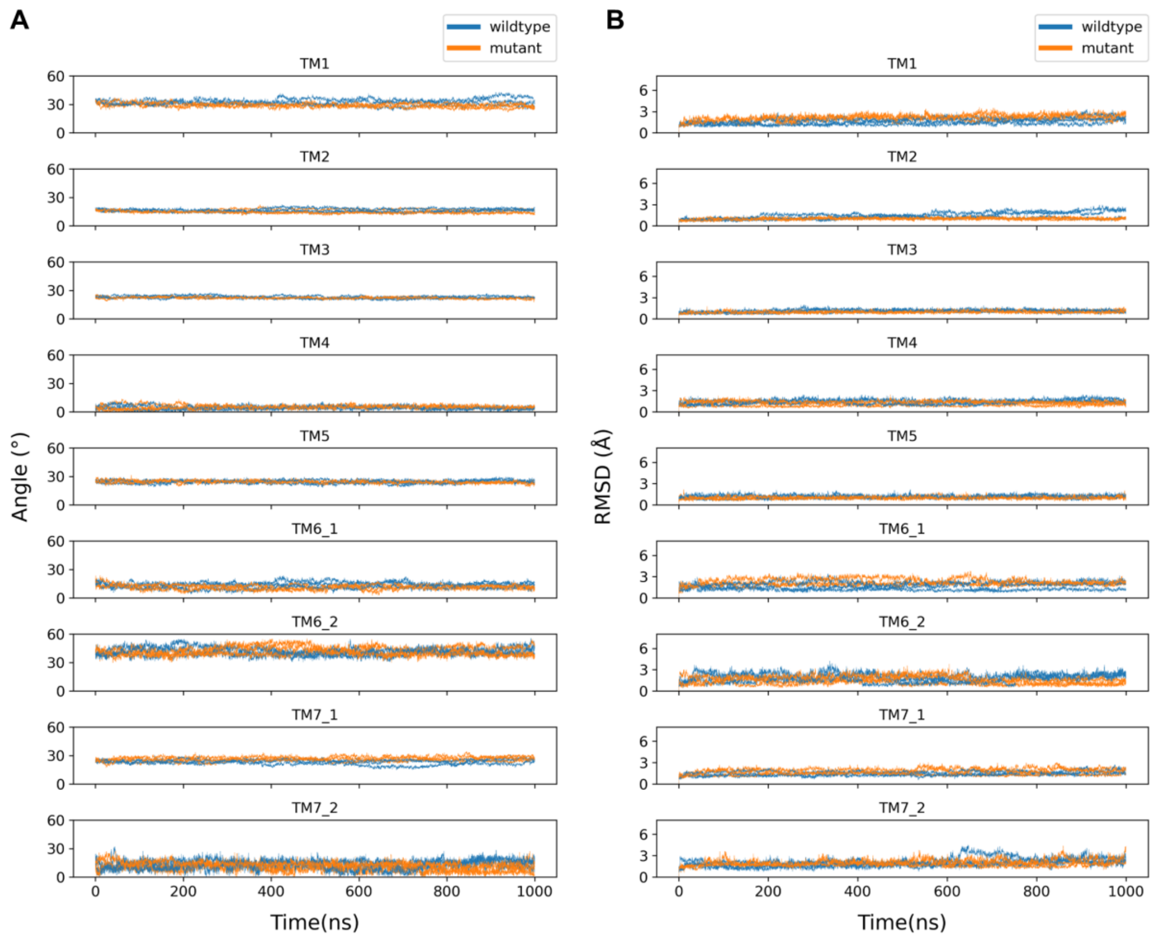


**Fig. S3. Time evolution of (A) angle and (B) RMSD for each helix in active state.** The wildtype and mutant systems are colored as blue and orange, respectively. The angle and RMSD of three replications are overlayed in the plot.

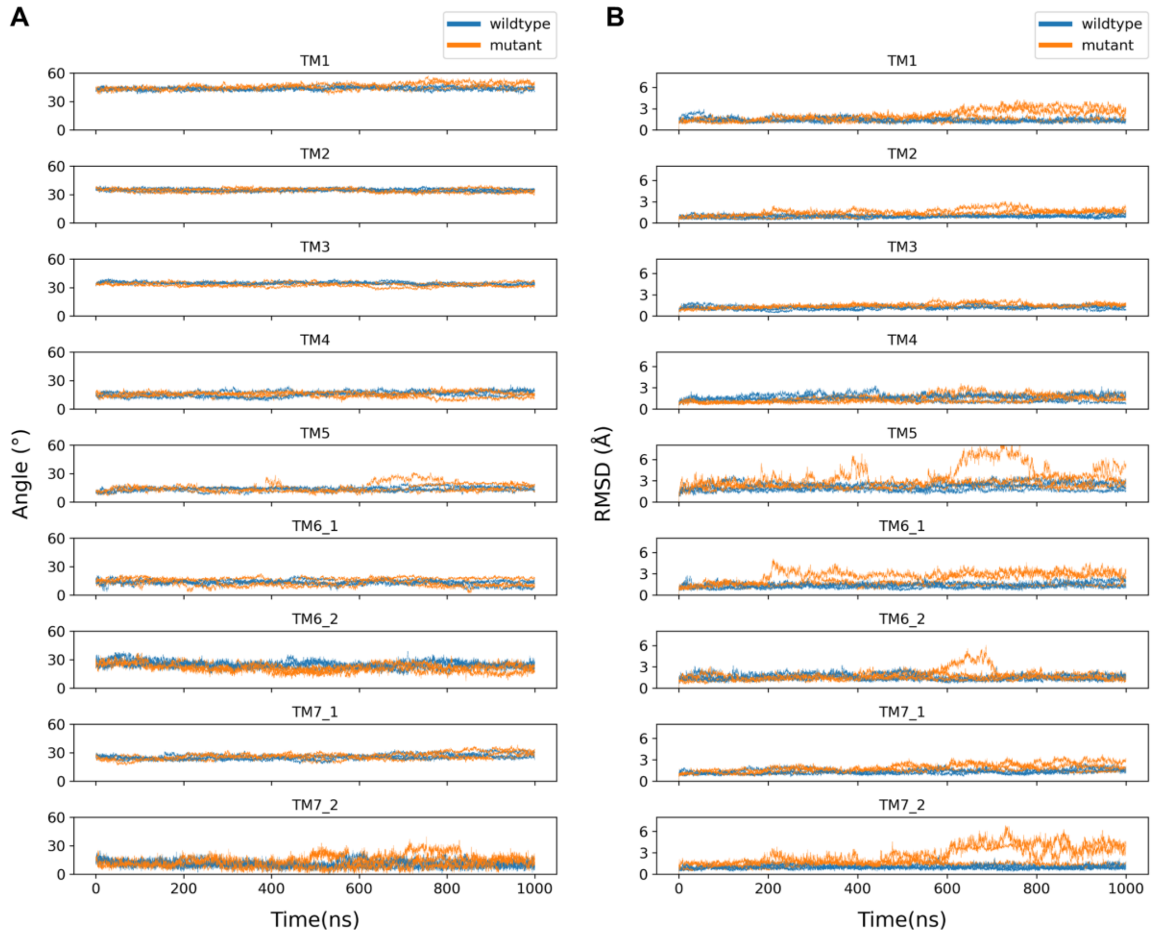


**Fig. S4. Time evolution of (A) angle and (B) RMSD for each helix in inactive state.** The wildtype and mutant systems are colored as blue and orange, respectively. The angle and RMSD of three replications are overlayed in the plot.

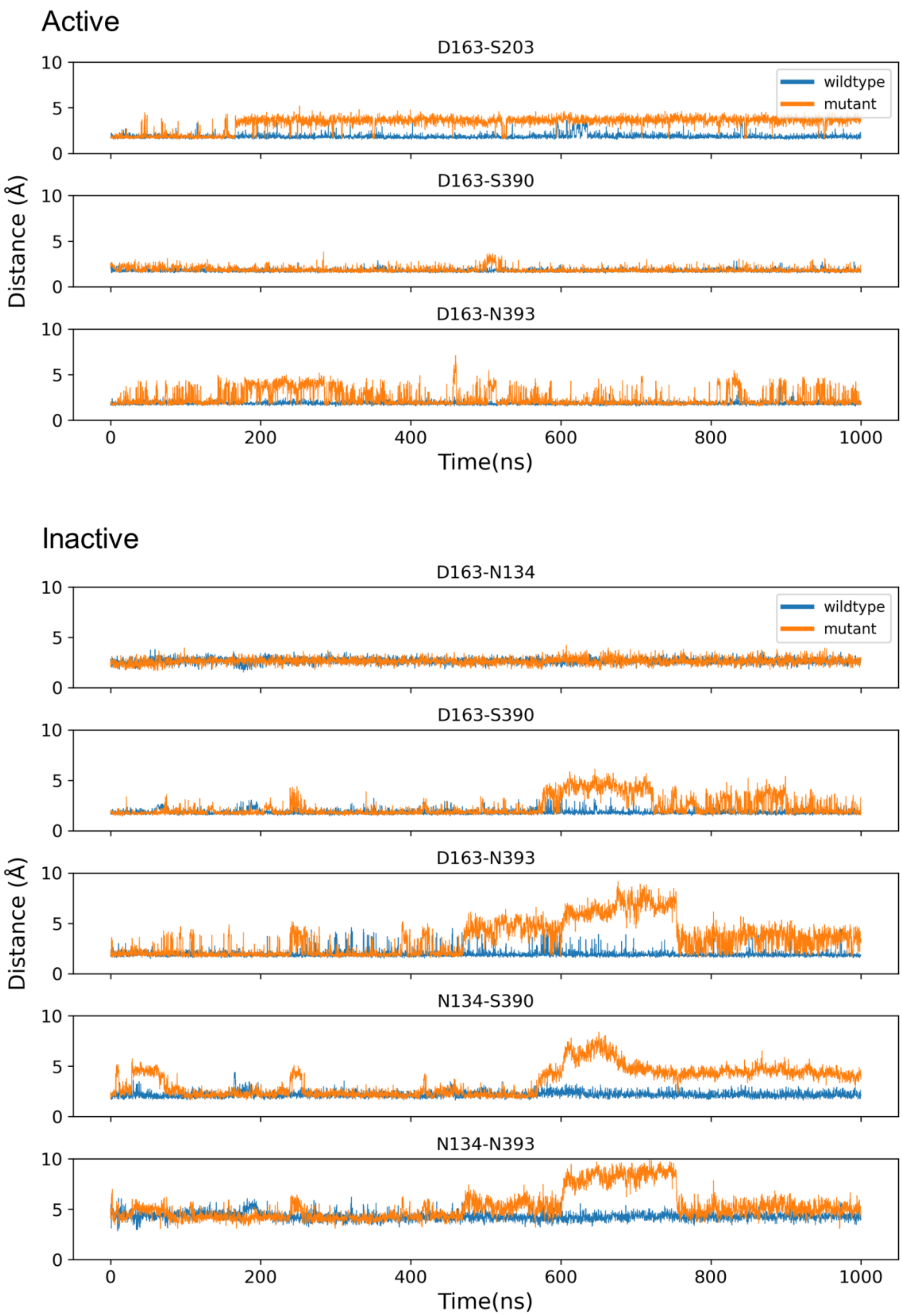


**Fig. S5. The hydrogen bond distances in Na+ pocket**. The wildtype and mutant CB1 are colored as blue and orange, respectively.

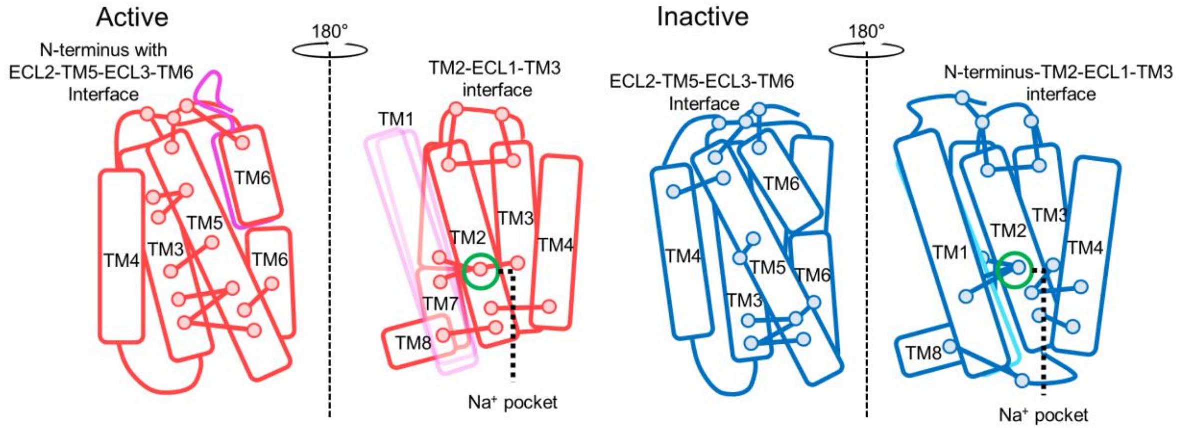


**Fig. S6. Schematic inter-helical interaction with relative helical position**. The active and inactive of wildtype CB1 structures are colored as red and blue and of mutant CB1 are magenta and cyan, respectively. Pairwise interactions are presented as solid lines, which are decreased after the mutation. Especially, the position of $Na^+$ pocket is marked as green circle.
